# Malnutrition in Surgical Wards: A Plea for Concern

**DOI:** 10.1155/2011/840512

**Published:** 2010-08-03

**Authors:** Offir Ben-Ishay, Haya Gertsenzon, Tanya Mashiach, Yoram Kluger, Irit Chermesh

**Affiliations:** ^1^Department of Surgery, Rambam Health Care Campus, Haifa 31096, Israel; ^2^Department of Quality Assurancey, Rambam Health Care Campus, Haifa 31096, Israel; ^3^Department of Gastroenterology, Rambam Health Care Campus, Haifa 31096, Israel

## Abstract

*Background*. Malnutrition in hospitalized patients is underdiagnosed, with 30 to 60% of patients admitted being malnourished. The objective of this study was to investigate the nutritional status of patients in a general surgery ward and to define the correlation between the risk of malnutrition and the hospital course and clinical outcome. *Study design*. The study group included 100 consecutive patients admitted to a general surgery ward who were ambulant and could undergo the Malnutrition Universal Screening Tool (MUST). *Results*. Thirty-two patients (33%) had aMUST score of 2 or higher, and were therefore defined at high-malnutrition risk. The patients at risk had longer hospitalization and worse outcome. The length of stay of the malnourished patients was significantly longer than that of patients without malnutrition risk (18.8 ± 11.5 vs. 7 ± 5.3 days, *P* = .003). Mortality in the high-risk group was higher overall, in hospital, and after six months and one year of followup. *Conclusions*. Medical personnel must be aware that malnutrition afflicts even patients whose background is not suggestive of malnutrition. Best results are achieved when cooperation of all staff members is enlisted, because malnutrition has severe consequences and can be treated easily.

## 1. Introduction

 In 1859, Florence Nightingale described soldiers in the Crimea hospital starving amongst plenty of food. Consequently, realizing the importance of nutrition to the well-being of patients, she suggested methods to remedy this problem. Still, more than one hundred years later, Hill et al. [1] found that 50% of surgical patients and 40% of medical patients were malnourished. In the majority of these patients, this risk increases during hospitalization [2]. As malnutrition in hospitalized patients is underdiagnosed, 70–80% of the malnourished are not identified as such. Therefore, no action is taken to treat their poor nutrition, and the diagnosis of malnutrition is not on their hospital discharge summary [3–5]. 

Malnutrition is a state of nutrient deficiency, a result of either inadequate nutrient intake or inability to absorb or use ingested nutrients. Professional organizations around the world highlight the frequent underreporting of malnutrition and advocate implementation of a simple and valid screening tool to identify patients at risk [6, 7]. For example, the European Society of Enteral and Parenteral Nutrition advocated nutritional screening because it could improve mental and physical function, reduce the number and severity of complications of disease or its treatment, accelerate recovery, save resources, and shorten hospital stay [7]. In view of the prevalence and deleterious consequences of malnutrition and the existence of an effective treatment, it is pragmatic to apply these recommendations and to introduce routine screening of patients for identification of patients at risk for malnutrition. For that, various screening tools exist, including the Nutritional Risk Screening (NRS-2002), Mini Nutritional Assessment (MNA) and the Malnutrition Universal Screening Tool (MUST) [7]. The different tools differ by the different parameters. Usually there is a tradeoff of complexity on the one hand and validity on the other hand. The NRS 2002 is a two-stage screening tool which takes into account various parameters such as severity of disease, age and could be applied to nonambulant patients. The MUST lacks these parameters but includes only three variables so it is very easy to perform. With this in mind, we chose the MUST for its validity, reliability, and simplicity even though it does not take into account some of the variables comprising other screening tools.

The objective of this study was to investigate the nutritional status of a cohort of sequential patients in a general surgery ward and to define the correlation between the risk of malnutrition and the hospital course and clinical outcome.

## 2. Methods

This prospective observational study was conducted in a 37-bed general surgery ward of Rambam Medical Center, a tertiary 970-bed hospital. MUST Assessment was performed as a part of routine work up, upon admission to our department. The study was approved by the local ethical committee. The study group included 100 consecutive patients in the department who were ambulant and could undergo the MUST evaluation on admission.

The MUST includes three variables: unintentional weight loss in the preceding 3 to 6 months, body mass index (BMI), and assessment of how the acute disease might affect the nutritional intake in the subsequent five days. Each is scored on a scale of 0, 1, or 2 and their sum categorizes the malnutrition risk as low (0), medium (1), or high (≥2). The MUST is easy, applicable, and reliable [8–10], and is used routinely to screen patients admitted to our department.

The nursing team received four-hour training in nutrition and nutritional screening. Continuing guidance was provided throughout the study by the nutrition team as part of the clinical service. The MUST was part of the anamnesis taken by the admitting nurse and was integrated into the patient's computed chart that is readily available to the treating physician.

Data collection included, in addition to the MUST score, demographic data and clinical information, hospital course and outcome, namely, age, gender, malignancy, elective or urgent admission, wound infection, the use of total parenteral nutrition or enteral nutrition during hospital stay, need for dialysis, and need for mechanical ventilation. The length of stay (LOS) was calculated, and in-hospital mortality, and at six months and one year of followup were recorded.

Data was processed using a statistical software package (SPSS v15). The Student's t-test and the Mann-Whitney test were used for comparison of continuous variables, as appropriate, and the Pearson's chi-square was applied for comparison of frequencies. Charslon comorbidity index was used to compare the group of patients at high risk for malnutrition to the group of patients not found at risk for malnutrition [11].

## 3. Results

One hundred consecutive patients underwent MUST screening, but 4 were excluded from the study because of subsequent missing data. Fifty four (56.25%) were males, and the median age of the study group was 54 years (18–94 years).

Thirty-two patients (33%) had a MUST score of 2 or higher, and were therefore categorized as having high-malnutrition risk. Fifty seven patients (~60%) were categorized in the low-malnutrition risk group with a MUST score of 0, and seven (~7%) patients had a medium risk for malnutrition, with a score of 1. There was no difference in the age or gender distribution between the high- and the low-malnutrition risk groups. The intermediate risk patients were combined with the low risk group, because no active treatment upon admission is advocated for either group.

Overall, the patients at risk had a longer hospitalization and worse outcome (Table 1). On univariate analysis, the LOS of the malnourished patients was significantly longer than that of patients without malnutrition risk (18.8 ± 11.5 versus 7 ± 5.3 days, resp., *P* = .003). Mortality in the high-risk group was higher overall, in hospital, and after six months and one year of followup. In hospital, three patients from the malnourished group died, while none from those not at risk (10% versus 0%, *P* = .03). One patient died due to sepsis complicating pancreatitis, one patient died of sepsis after traumatic perforation of the rectum, and one patient died of multiorgan failure following mesenteric ischemia. Only one no-risk patient died during the year of followup (in the first six months) compared to four high-risk patients (Table 1). Multivariate analysis could not be done due to the small number of patients overall and deceased. Charlson comorbidity index did not differ significantly between the group of the patients at high risk for malnutrition and the group of patients who was not found to be at risk for malnutrition (maybe because of the small numbers) Table 2.

Overall, more than double, 16% versus 8% (*P* = .3), of the patients in the malnourished group received advanced nutritional therapy (parenteral or enteral). More of the malnourished patients were admitted on an urgent basis directly from the emergency department (68.2% versus 53.2%), but this did not reach statistical significance. No difference was found between rates of dialysis, need for mechanical ventilation, and rate of wound infection.

## 4. Discussion

Malnutrition has been recognized as a major problem in surgical patients for many years. Hill et al. [1] recognized malnutrition in surgical patients as a leading cause of morbidity and mortality. Frequently unnoticed protein and energy malnutrition can cause anemia, hypoalbuminemia, vitamin deficiencies, and weight loss [1]. Malnutrition is also responsible for impaired immunity [11], and could result in increased complications such as pressure ulcers, delayed wound healing, and increased risk of infections, impaired muscular and respiratory functions as well as increased mortality [8, 12–15].

Nutritive treatment can be enriched by oral diet, or provided through enteral or parenteral route. All these alternatives are efficient in lowering complications and mortality in hospitalized malnourished patients [16, 17]. Enteral nutrition is preferable because there are fewer related complications and it is more cost-effective than parenteral nutrition. When the enteral route is unavailable or fails, parenteral nutrition is indicated; yet, either requires close followup.

Stratton [6] performed a meta-analysis on patients receiving routine care and patients receiving multinutrient oral or tube supplements, and found that those receiving supplements had a significantly lower complication and mortality rates. Supplementation was also associated with reduction in sepsis, wound infection, pneumonia, and decubitus ulcers [6].

Analysis of the risk of mortality and the LOS in a group of patients screened by the MUST found that patients at medium risk (MUST score of 1) had increased mortality, similar to that of high-malnutrition risk patients [18]. However, while our study group includes 96 consecutive patients and provided reliable and unbiased results when applying the MUST as a screening tool, it was not large enough to allow comparison of various subgroups of patients, such as those with malignant or benign diseases, young versus old, and the outcome of patients in the medium risk group. The small number of patients in the intermediate group rises questions on the one hand it could be that the MUST is inadequate as a screening tool for some of the patients; on the other hand this dichotomy could imply that the MUST can differentiate between a high-risk group and the low-risk group very effectively.

One third of the patients in our study were at risk of malnutrition, confirming that malnutrition in hospitalized patients is prevalent. Malnourished patients run a complicated course of hospitalization with worse outcome, longer LOS, and higher mortality rates, and indeed, we found that high-risk patients in our cohort had longer LOS and increased mortality in-hospital and throughout the first year of followup. This data is in line with the data from the European NutritionDay study. This is a cross-sectional study, which takes place every year since 2006. More than 24,000 patients took part in this audit over the years. On the NutritionDay audit, information regarding nutritional status is collected from the treating staff and the patients themselves. In 2010, 8155 patients from a wide range of departments took part in the NutritionDay. Over 40% of the patients lost weight before admission only 50% ate normally on the week before the NutritionDay was held. On NutritionDay itself, only 44% of the patients ate everything that was served for lunch. Data analysis from previous years showed that decreased food intake on NutritionDay or during the previous week was associated with an increased risk of dying, even after adjustment for various patient and disease-related factors. Adjusted hazard ratio for dying when eating about a quarter of the meal on NutritionDay was 2.10 (1.53–2.89) when eating nothing 3.02 (2.11–4.32) [19]. No information regarding food intake was collected in this study. Six-year survival was assessed by Sullivan et al. in a group of 350 patients discharged from the hospital; the variable most strongly associated with mortality was “nutrition risk” [20].

Clinicians need to be able to identify patients who are malnourished or at risk of malnutrition, especially since nutrition treatment protocols are effective, and most of the patients could be treated according to them. A multidisciplinary nutritional support team is beneficial when treating complicated patients [21]. In medical centers where such a team had been established, optimal nutritional care was provided resulting in improved outcome [3, 22]. It is also prudent to identify patients at malnutrition risk, and the MUST is an efficient mean for accomplishing this [23]. We have concluded that screening of patients for malnutrition is mandatory, and the MUST is efficient and simple to apply and interpret. After identification of patients at risk for malnutrition, assessment should be performed. Malnourished patients should be treated by an efficient method. So far, the best-proven treatment modality is artificial nutrition. Enteral nutrition is the preferred route, but the enteral route is unavailable; parenteral nutrition should be given promptly.

The findings of our study reflect the urgent need for awareness of physicians, nursing staff, and dieticians to the problem of malnutrition in surgical departments. Best results are achieved when cooperation of all staff members is enlisted because malnutrition has severe consequences and is easily treated. Future randomized prospective controlled trials are advocated.

## 5. Conclusion

Malnutrition is prevalent and has association with longer length of stay and higher mortality rates. Identifying patients at risk is easy and feasible. Screening for malnutrition should be performed on a routine basis using a validated tool.

Limitations of the study: this is a small study with a limited number of patients. Further studies preferably prospective randomized studies are warranted so that optimal treatment protocols are set up.

## Figures and Tables

**Table 1:** Patients characteristics, hospitalization, and outcome.

	High risk group	No risk group	*P*
Patients	32 (33.33%)	64 (66.67%)	
Median Age (y)	57 (24–94)	54 (19–90)	NS
Gender (male)	17 (53.12%)	35 (54.68%)	NS
Admission- emergency (versus elective)	22 (68.8%)	34 (53.1%)	.3
Malignancy (versus benign)	14 (43.72%)	12 (18.75%)	.02
Surgery performed	19 (59.37%)	38 (59.37%)	.8
LOS (d)*	18.8 ± 11.5	7 ± 5.3	.003
Nutritional therapy	15.6%	7.9%	.3
Mortality			
In hospital	3 (9.4%)	0 (0%)	.017
Cumulative 6 months	6 (18.8%)	1 (1.6%)	.006
Cumulative 12 months	7 (21.9%)	1 (1.6%)	.002

*Mean ± SD.

**Table 2:** Charlson comorbidity index.

		MUST			
		0 + 1		2+	
		64 (*n*)	%	32 (*n*)	%
Charlson index score	0	33	51.6	12	37.5
	1	15	23.4	6	18.8
	2	6	9.4	6	18.8
	3	6	9.4	3	9.4
	4+	4	6.3	5	15.6

Mann-Whitney U *P* = .08.

## List of Abbreviations

MUST: Malnutrition Universal Screening Tool
BMI: Body Mass Index
LOS: Length of Stay.
